# Treatment adequacy of anxiety disorders among young adults in Finland

**DOI:** 10.1186/s12888-016-0766-0

**Published:** 2016-03-15

**Authors:** Teija Kasteenpohja, Mauri Marttunen, Terhi Aalto-Setälä, Jonna Perälä, Samuli I. Saarni, Jaana Suvisaari

**Affiliations:** Department Health, Mental Health Unit, National Institute for Health and Welfare, P.O. Box 30, Helsinki, 00271 Finland; Adolescent Psychiatry, University of Helsinki and Helsinki University Hospital, Helsinki, Finland; The Social Insurance Institute, Helsinki, Finland; Psychiatry, University of Helsinki and Helsinki University Hospital, Helsinki, Finland; Turku University Hospital and University of Turku, Turku, Finland; Department of Social Psychiatry, School of Public Health, University of Tampere, Tampere, Finland; Faculty of Medicine, University of Helsinki, Helsinki, Finland

**Keywords:** Anxiety disorders, Epidemiology, Quality of care, Young adults

## Abstract

**Background:**

Anxiety disorders are common in early adulthood, but general population studies concerning the treatment adequacy of anxiety disorders taking into account appropriate pharmacological and psychological treatment are scarce. The aims of this study were to examine treatments received for anxiety disorders in a Finnish general population sample of young adults, and to define factors associated with receiving minimally adequate treatment and with dropping out from treatment.

**Methods:**

A questionnaire containing several mental health screens was sent to a nationally representative two-stage cluster sample of 1894 Finns aged 19 to 34 years. All screen positives and a random sample of screen negatives were invited to a mental health assessment including a SCID interview. For the final diagnostic assessment, case records from mental health treatments for the same sample were obtained. This article investigates treatment received, treatment adequacy and dropouts from treatment of 79 participants with a lifetime anxiety disorder (excluding those with a single specific phobia). Based on all available information, receiving antidepressant or buspirone medication for at least 2 months with at least four visits with any type of physician or at least eight sessions of psychotherapy within 12 months or at least 4 days of hospitalization were regarded as minimally adequate treatment for anxiety disorders. Treatment dropout was rated if the patient discontinued the visits by his own decision despite having an adequate treatment strategy according to the case records.

**Results:**

Of participants with anxiety disorders (excluding those with a single specific phobia), 41.8 % had received minimally adequate treatment. In the multivariate analysis, comorbid substance use disorder was associated with antidepressant or buspirone medication lasting at least 2 months. Those who were currently married or cohabiting had lower odds of having at least four visits with a physician a year. None of these factors were associated with the final outcome of minimally adequate treatment or treatment dropout. Participants with comorbid personality disorders received and misused benzodiazepines more often than others.

**Conclusions:**

More efforts are needed to provide adequate treatment for young adults with anxiety disorders. Attention should be paid to benzodiazepine prescribing to individuals with personality disorders.

**Electronic supplementary material:**

The online version of this article (doi:10.1186/s12888-016-0766-0) contains supplementary material, which is available to authorized users.

## Background

Anxiety disorders are among the most prevalent mental disorders worldwide [1–3], and they typically affect young people [1]. In the World Mental Health Survey, their lifetime prevalence varied from 4.8 % in China to 31.0 % in the United States [1]. The impact of chronic anxiety disorders on quality of life is significant: among Finnish Health 2000 survey participants aged over 30 years, chronic anxiety disorders and dysthymia were associated with the largest loss of HRQoL (health-related quality of life) among mental disorders [4]. When 29 chronic conditions were studied, anxiety disorders had the second largest negative impact on HRQoL just after Parkinson’s disease [5].

According to previous studies, anxiety disorders in the general population tend to be under-diagnosed [6] and under-treated [7–9] and individuals with anxiety disorders often experience long delays in seeking treatment [10, 11]. The prevalence of service use for mental health problems during the last 12 months varied from 20.6 to 44.9 % among respondents with anxiety disorders [7, 8, 12–15].

General population studies concerning the treatment adequacy of anxiety disorders taking into account appropriate pharmacological and psychological treatment are scarce, while the criteria for minimally adequate treatment have likewise varied [7, 15–17]. In most of the studies, anxiolytic medication was considered an adequate treatment, with benzodiazepines still being widely used by clinicians in spite of the controversy around possible physical dependency and an association with worse long-term outcomes [18, 19].

Mental health treatment dropout is a common problem [20–23] that may negatively affect the treatment outcome [24] and indicate poor functioning of the health care system [25]. Establishing which patient related factors affect discontinuation would provide important information for improving mental health services.

This study uses a nationwide, representative population-based sample of young Finnish adults aged 20–34 years. The aims of this study were to describe the frequency and the type of treatment received for anxiety disorders and to describe treatment adequacy and discontinuation and socio-demographic and clinical features affecting them among young adults.

## Methods

### Sample

Data were derived from the Mental Health in Early Adulthood in Finland (MEAF) study. The methods have been reported in detail elsewhere [26, 27]. Briefly, MEAF was based on a nationally representative two-stage cluster sample (*n* = 1894) of young adults aged 18–29 years taken from the Health 2000 study, which was a comprehensive health survey. The original young adult assessment was carried out in 2001. There were also questions related to mental health in the Health 2000 young adult protocol, though a structured diagnostic interview was not conducted [28–30]. Therefore, a substudy focusing on mental health (MEAF) was carried out [26, 27].

The study flow of the MEAF study is presented in the Additional file 1. A questionnaire including several scales assessing mental health and substance use was mailed 2–4 years after the original study to all living members of the sample, excluding those who had refused further contacts. All screen-positive and a random subsample of screen-negative participants were invited to the mental health interview. Information from the Finnish National Hospital Discharge Register (NHDR) was used to identify all persons who had received hospital treatment due to any mental disorder [International Classification of Diseases (ICD)-10 section F, ICD-8 and ICD-9 290–319] and these were also asked to participate in the interview. The screening procedure has been described in detail previously [26, 27].

### Ethics, consent and permissions

The ethics committees of the National Institute for Health and Welfare and the Hospital District of Helsinki and Uusimaa approved the Health 2000 survey and the MEAF reassessment. Participants gave written informed consent [26, 28].

### Mental health assessment

The mental health interview was conducted using the research version of the Structured Clinical Interview for DSM-IV (SCID-I) [31], which included the SCID Screening module at the beginning to enhance reliability [32], as well as the sections on mood, psychotic, substance use, anxiety and eating disorders. The interview included questions on socio-demographic factors and treatment received for mental health problems. Experienced research nurses or psychologists attended a 1-week training course and had regular follow-up sessions to prepare them to carry out the interviews, which were then reviewed together with a psychiatrist.

### Final diagnostic assessment

For the final diagnostic assessment, all case records from hospital and outpatient treatment contacts were obtained with the participant’s approval. The Finnish Ministry of Social Affairs and Health gave permission to view the case records of non-participants, excluding those who had refused any participation in the Health 2000 study. Four experienced clinicians (J.S., T.A.-S., S.S., J.P.) made the final best-estimate diagnoses using DSM-IV-TR criteria, based on all available systematically evaluated information from the interview and/or case records. Disorders not covered by SCID-I, e.g. personality disorders, were also evaluated [26].

As reported previously, the lifetime prevalence of anxiety disorders (i.e. panic disorder, agoraphobia, obsessive-compulsive disorder, social phobia, specific phobia, post-traumatic stress disorder, generalized anxiety disorder, anxiety disorder NOS) in this sample was 12.6 % [26]. This paper investigates this subgroup of 92 participants after excluding those with a diagnosis of schizophrenia spectrum psychotic disorders (3 persons).

### Use of mental health services and treatment received

Information on treatment received for anxiety disorders was gathered from mental health interviews and case records. We evaluated the visits and medication use for anxiety disorders during the 12 months that a participant was most intensively treated. All data were collected on both the most recent and the most intensive treatment period for anxiety disorder. However, the present paper focuses on the most recent treatment period, since our aim was to investigate the typical and the most recent functioning of the health care system. Results concerning the most intensive treatment period are available in Additional files 2, 3, 4, 5 and 6.

It has been previously observed that patients with isolated specific phobias very rarely seek professional help [33]. Also in our sample, participants with specific phobia more often had no treatment than others (Table 3). Due to the lesser clinical significance and a different treatment model [34, 35], we excluded those whose only anxiety disorder was specific phobia (13 subjects). The final analysis of treatments received therefore included 79 participants.

### Criteria for minimally adequate treatment

Criteria for minimally adequate treatment were determined according to evidence-based guidelines [34, 36–42] and were the same as those used in the European ESEMeD and the National Comorbidity Survey Replication [43, 44], except for hospital treatment and anxiolytic medication. As the benefits and disadvantages of long-lasting use of benzodiazepines are controversial, we regarded only the use of antidepressant or buspirone for at least 2 months as an appropriate pharmacotherapy, and studied the use and misuse of benzodiazepines separately. Hospitalizations have not been used as an adequacy criterion in previous surveys. However, because we had accurate information about hospitalizations based on the national hospital discharge register and corresponding case records, we used for at least 4 days of hospitalizations for anxiety symptoms as an adequacy criterion. Based on the information in the case records and the fact that evaluation period in Finland is 4 days, we considered it sufficient to assess the diagnosis as well as plan and start a treatment. On the other hand, this time limit excluded for example short visits in the emergency department.

Guideline-concordant treatment was defined as follows:

1. Pharmacotherapy: use of antidepressant or buspirone for at least 2 months and at least four visits within 12 months with a physician for anxiety disorders. Included in visits were telephone consultations between patient and physician, as well as consultations between health care professionals and the treating physician concerning the patient’s treatment.
2. Psychotherapy: at least eight sessions within 12 months with a psychiatrist, psychologist or psychotherapist in any settings or with another professional in a psychiatric clinic for anxiety disorders.
3. Hospital treatment: at least 4 days of hospitalization for anxiety disorders.

A final outcome of minimally adequate treatment was recorded if pharmacotherapy or psychotherapy or hospital treatment was defined to be adequate.

Benzodiazepine misuse was recorded if a subject had a diagnosis for benzodiazepine abuse or dependence or had told about misuse in the interview or during the treatment period. We took into account only misuse during the index treatment period.

Treatment dropout was recorded when the treatment strategy was assessed to be adequate according to the case records but the patient discontinued the visits by his own volition.

### Sociodemographic, disorder-specific and comorbidity factors

We studied the relationship between socio-demographic factors and treatment using the following variables: gender, marital status, age at the time the MEAF-questionnaire was sent, basic education and current employment. Only the effect of basic education was examined, since some of the younger members of the cohort had not yet finished their vocational or higher education.

The effect of comorbid psychiatric disorders on treatment received, treatment adequacy and dropouts from treatment was studied using the following disorder categories: mood disorders, substance use disorders, personality disorders and other disorders (psychotic, eating, sleeping, adjustment and impulse control disorders). We also studied if the number of different anxiety disorders (one or more) affected treatment.

### Statistical analysis

Standard statistical methods were used to generate descriptive statistics. Since the analysis was limited to the subgroup of participants with anxiety disorders only, we did not use survey weights in the analysis. We analyzed the associations of socio-demographic factors and comorbid psychiatric disorders with the treatments received. The relationship of all these variables was examined separately for different components of care, i.e. pharmacotherapy with antidepressants and buspirone, benzodiazepine use and misuse, physician visits, psychotherapy, treatment adequacy and dropout from treatment. Differences were tested using the *χ*^2^ or Fisher’s exact tests. Logistic regression analysis was used to identify variables independently associated with the type and adequacy of treatment. Gender, basic education, marital status and different comorbid disorders were entered simultaneously into a logistic regression model to explore the factors affecting treatment. All statistical tests were two-tailed, and values of *p* < 0.05 were considered statistically significant. We used the SAS 9.3 statistical package in the analyses.

## Results

### Participants

Male participants with any lifetime diagnosis of anxiety disorder numbered 26 (28.3 %) and female participants 66 (71.7 %). There were no statistically significant differences in socio-demographic factors between genders, but men had more substance use disorders (Table 1).

**Table 1:** Socio-demographic factors and comorbid psychiatric disorders of participants with a lifetime diagnosis of anxiety disorder

		All		Men		Women		
		100.0 %	*N* = 92	28.3 %	*N* = 26	71.7 %	*N* = 66	
Variable	Category	%	N	%	N	%	N	p*
Age	<25 years	19.6	18	15.4	4	21.2	14	
	25–29 years	40.2	37	42.3	11	39.4	26	
	≥30 years	40.2	37	42.3	11	39.4	26	0.818
Basic education	Less than high school	52.8	47	69.6	16	47.0	31	
	High school	47.2	42	30.4	7	53.0	35	0.062
Married or cohabiting	Yes	66.3	59	56.5	13	69.7	46	
	No	33.7	30	43.5	10	30.3	20	0.250
Current employment	Employed	56.2	50	69.6	16	51.5	34	
	Student^c^	18.0	16	21.7	5	16.7	11	
	Unemployed	11.2	10	8.7	2	12.1	8	
	Other^a^	14.6	13	0.0	0	19.7	13	0.077**
Comorbid mood disorder	Yes	58.7	54	61.5	16	57.6	38	
	No	41.3	38	38.5	10	42.4	28	0.728
Comorbid personality disorder	Yes	21.7	20	30.8	8	18.2	12	
	No	78.3	72	69.2	18	81.8	54	0.188
Comorbid substance abuse or dependence (any substance)	Yes	28.3	26	57.7	15	16.7	11	
	No	71.7	66	42.3	11	83.3	55	**<0.0001**
Comorbid other disorder^b^	Yes	18.5	17	7.7	2	22.7	15	
	No	81.5	75	92.3	24	77.3	51	0.137

^a^Of the other group, 10 (76.9 %) were at home taking care of household and family members, 2 (15.4 %) were on disability pension or sick leave and 1 (7.7 %) was on return to work trial after sickness absence

^b^Psychotic, eating, sleeping, adjustment or impulse control disorder lifetime

^c^Students in vocational schools or universities

*The *p*-values indicate a significance of the difference between genders in the distribution of each category tested by *χ*2- or Fisher’s exact test. *P*-values < 0.05 in boldface

**Fisher’s exact test was used in the analysis

The most common anxiety disorder was panic disorder, affecting 27.2 % of participants, followed by social phobia (25.0 %), anxiety disorder NOS (25.0 %) and specific phobia (20.7 %). Less common were agoraphobia without panic disorder (7.6 %), posttraumatic stress disorder (6.5 %), obsessive-compulsive disorder (5.4 %), and generalized anxiety disorder (3.3 %) (Table 2).

**Table 2:** The percentage distribution of different anxiety disorders among the participants

	All (*N* = 92)		Men (*N* = 26)		Women (*N* = 66)		p*
Variable	%	N	%	N	%	N	
Panic disorder with agoraphobia	20.7	19	23.1	6	19.7	13	0.718
Panic disorder without agoraphobia	6.5	6	3.9	1	7.6	5	0.672**
Agoraphobia without panic disorder	7.6	7	11.5	3	6.1	4	0.399**
Social phobia	25.0	23	38.5	10	19.7	13	0.061
Obsessive-compulsive disorder	5.4	5	3.9	1	6.1	4	1.000**
Posttraumatic stress disorder	6.5	6	0.0	0	9.1	6	0.179**
Generalized anxiety disorder	3.3	3	3.9	1	3.0	2	1.000**
Specific phobia	20.7	19	15.4	4	22.7	15	0.433
Anxiety disorder NOS	25.0	23	23.1	6	25.8	17	0.789

*The *p*-values indicate a significance of the difference between genders in the distribution of each category tested by *χ*2- or Fisher’s exact test. *P*-values < 0.05 in boldface

**Fisher’s exact test was used in the analysis

Participants with specific phobia had statistically significantly more often no treatment than others with anxiety disorder, whereas participants with anxiety disorder NOS were less often without treatment (Table 3). After excluding those whose only anxiety disorder was specific phobia, we had 22 male (27.9 %) and 57 (72.2 %) female participants.

**Table 3:** The percentage distribution of treatment contacts among participants with different anxiety disorders

		Treatment		No treatment		
Variable	Category	%	N	%	N	p*
All		61.2	57	38.0	35	
Gender	Men	65.4	17	34.6	9	
	Women	60.6	40	39.4	26	0.671
Panic disorder with agoraphobia		79.0	15	21.1	4	0.087
Panic disorder without agoraphobia		66.7	4	33.3	2	1.000**
Agoraphobia without panic disorder		42.9	3	57.1	4	0.421**
Social phobia		65.2	15	34.8	8	0.710
Obsessive-compulsive disorder		60.0	3	40.0	2	1.000**
Posttraumatic stress disorder		66.7	4	33.3	2	1.000**
Generalized anxiety disorder		66.7	2	33.3	1	1.000**
Specific phobia		21.1	4	79.0	15	**<0.0001**
Anxiety disorder NOS		91.3	21	8.7	2	**0.001**

*The *p*-values indicate a significance of the difference between categories in the distribution of treatment tested by *χ*2- or Fisher’s exact test. *P*-values < 0.05 in boldface

**Fisher’s exact test was used in the analysis

### Treatment received and treatment adequacy

After excluding those with a single specific phobia, 70.9 % of participants had had some kind of contact with the health care system for their anxiety disorders. More than two thirds had visited a physician, and 36.4 % had had at least four visits within 12 months. More than half had been prescribed an antidepressant or buspirone, and 43.4 % had used them for at least 2 months. Guideline-concordant pharmacotherapy including also physician visits was received by 26.6 % of participants. 57.9 % of subjects had attended psychotherapy sessions, more than a third at least eight times within 12 months. Adequate hospital treatment was received by 10.1 % of the sample (8 participants). A total of 41.8 % of subjects had received minimally adequate treatment. 11.0 % of participants had discontinued their visits despite of a proper treatment plan (Table 4). Benzodiazepines were prescribed to 34.6 % of participants, and 5.1 % had misused them during the most recent treatment period (Table 6).

**Table 4:** Sociodemographic factors, treatments received and dropouts during the most recent treatment episode for anxiety disorders^h, i^

			Pharmacotherapy				Visits with a physician				Guideline concordant pharmacotherapy^c^		Sessions of psychotherapy / a year				Minimally adequate treatment^e^		Treatment dropout^f^	
			Any^a^		≥2 months		Any^b^		≥4 times				Any^d^		≥8 times					
Variable	Category		%	N	%	N	%	N	%	N	%	N	%	N	%	N	%	N	%	N
All			51.3	40	43.4	33	68.8	53	36.4	28	26.6	21	57.9	44	34.2	26	41.8	33	11.0	8
Gender	Male		59.1	13	54.6	12	71.4	15	42.9	9	36.4	8	71.4	15	33.3	7	45.5	10	9.5	2
	Female		48.2	27	38.9	21	67.9	38	33.9	19	22.8	13	52.7	29	34.6	19	40.4	23	11.5	6
		p*	0.387		0.212		0.763		0.468		0.222		0.140		0.921		0.680		1.000	
Agegroup	<25 years		66.7	10	60.0	9	78.6	11	42.9	6	40.0	6	71.4	10	57.1	8	60.0	9	7.7	1
	25–29 years		45.5	15	39.4	13	61.8	21	29.4	10	23.5	8	51.5	17	12.1	4	29.4	10	12.5	4
	≥30 years		50.0	15	39.3	11	72.4	21	41.4	12	23.3	7	58.6	17	48.3	14	46.7	14	10.7	3
		p*	0.389		0.352		0.453		0.527		0.426		0.447		**0.002**		0.107		1.000^g^	
Basic	Less than high school		48.7	18	36.1	13	67.6	25	35.1	13	23.7	9	61.1	22	36.1	13	44.7	17	18.2	6
education	High school		50.0	19	46.0	17	68.4	26	34.2	13	26.3	10	51.4	19	32.4	12	36.8	14	5.4	2
		p*	0.907		0.393		0.937		0.933		0.791		0.401		0.741		0.484		0.136^g^	
Current	Employed		42.5	17	38.5	15	62.5	25	22.5	9	15.0	6	47.5	19	17.5	7	30.0	12	13.5	5
employment	Student		71.4	10	57.1	8	71.4	10	57.1	8	42.9	6	69.2	9	53.9	7	50.0	7	14.3	2
	Unemployed		44.4	4	44.4	4	62.5	5	62.5	5	44.4	4	66.7	6	55.6	5	66.7	6	12.5	1
	Other		50.0	6	27.3	3	84.6	11	30.8	4	23.1	3	63.6	7	54.6	6	46.2	6	0.0	0
		p*	0.331^g^		0.491^g^		0.517^g^		**0.033** ^g^		0.084^g^		0.445^g^		**0.008** ^g^		0.167		0.689^g^	
Married or	No		63.0	17	46.2	12	80.8	21	53.9	14	33.3	9	69.2	18	46.2	12	55.6	15	12.0	3
cohabiting	Yes		41.7	20	38.3	18	61.2	30	24.5	12	20.4	10	48.9	23	27.7	13	32.7	16	11.1	5
		p*	0.077		0.514		0.084		**0.011**		0.213		0.094		0.111		0.052		1.000^g^	

^a^Antidepressant or buspirone prescribed

^b^At least 1 visit with a physician a year

^c^Antidepressant or buspirone used for at least 2 months + 4 visits with a physician a year

^d^At least 1 session of psychotherapy a year

^e^Antidepressant or buspirone used for at least 2 months + at least 4 visits with a physician a year or at least 8 sessions of psychotherapy a year or a hospitalization for anxiety disorders lasting for at least 4 days

^f^A participant discontinued the visits despite adequate treatment plan

^g^Fisher’s exact test was used in the analysis

^h^Participants with a single specific phobia were excluded

^i^A bivariate analysis

*The *p*-values indicate a significance of the difference between categories in a distribution of treatments and dropout tested by *χ*2- or Fisher’s exact test. *P*-values < 0.05 in boldface

### Socio-demographic factors, treatment received and dropouts

Those who were currently employed had less often had four visits with a physician and eight psychotherapy sessions a year than other groups in the bivariate analysis (Table 4).

Marital status was statistically significantly associated with appointments with physicians: those who were currently cohabiting had less often had at least four visits with a physician a year in the bivariate analysis (Table 4).

Age at the time of the study had an association with psychotherapy sessions: the middle age group had had less often than others at least 8 sessions of psychotherapy a year (Table 4).

In our study group, 11.0 % of participants dropped out of treatment. No associations were found between socio-demographic factors and dropouts (Table 4).

### Comorbid psychiatric disorders, treatment received and dropouts

In the bivariate analysis, comorbid substance use disorder was related to antidepressant or buspirone medication for at least 2 months and receiving benzodiazepines (Tables 5 and 6). There was also an association between comorbid substance use disorder and receiving psychotherapy (Table 5).

**Table 5:** Comorbid disorders, treatments received and dropouts during the most recent treatment episode for anxiety disorders^i, j^

			Pharmacotherapy				Visits with a physician				Guideline concordant pharmacotherapy^c^		Sessions of psychotherapy / a year				Minimally adequate treatment^e^		Treatment dropout^f^	
			Any^a^		≥2 months		Any^b^		≥4 times				Any^d^		≥8 times					
Variable	Category		%	N	%	N	%	N	%	N	%	N	%	N	%	N	%	N	%	N
Comorbid	Yes		54.2	26	45.8	22	71.7	33	41.3	19	31.3	15	58.3	28	35.4	17	45.8	22	13.6	6
mood	No		46.7	14	39.3	11	64.5	20	29.0	9	19.4	6	57.1	16	32.1	9	35.5	11	6.9	2
disorders		p*	0.519		0.579		0.502		0.272		0.243		0.919		0.772		0.362		0.465^g^	
Comorbid	Yes		65.2	15	63.6	14	77.3	17	50.0	11	39.1	9	78.3	18	39.1	9	47.8	11	4.8	1
substance use	No		45.5	25	35.2	19	65.5	36	30.9	17	21.4	12	49.1	26	32.1	17	39.3	22	13.5	7
disorder		p*	0.111		**0.023**		0.312		0.116		0.106		**0.018**		0.552		0.484		0.425^g^	
Comorbid	Yes		68.4	13	52.6	10	73.7	14	57.9	11	40.0	8	73.7	14	52.6	10	60.0	12	15.8	3
personality	No		45.8	27	40.4	23	67.2	39	29.3	17	22.0	13	52.6	30	28.1	16	35.6	21	9.3	5
disorder		p*	0.086		0.350		0.599		**0.025**		0.116		0.108		0.051		0.056		0.421^g^	
Comorbid other	Yes		56.3	9	40.0	6	56.3	9	56.3	9	37.5	6	50.0	8	43.8	7	50.0	8	6.3	1
disorder^h^	No		50.0	31	44.3	27	72.1	44	31.2	19	23.8	15	60.0	36	31.7	19	39.7	25	12.3	7
		p*	0.656		0.765		0.239^g^		0.063		0.343^g^		0.472		0.365		0.455		0.676^g^	
More than 1	Yes		46.7	7	40.0	6	64.3	9	35.7	5	25.0	4	60.0	9	40.0	6	37.5	6	0.0	0
anxiety	No		52.4	33	44.3	27	69.8	44	36.5	23	27.0	17	57.4	35	32.8	20	42.9	27	13.6	8
disorder		p*	0.691		0.765		0.753^g^		0.956		1.000^g^		0.854		0.598		0.698		0.340^g^	

^a^Antidepressant or buspirone prescribed

^b^At least 1 visit with a physician a year

^c^Antidepressant or buspirone used for at least 2 months + 4 visits with a physician a year

^d^At least 1 session of psychotherapy a year

^e^Antidepressant or buspirone used for at least 2 months + at least 4 visits with a physician a year or at least 8 sessions of psychotherapy a year or a hospitalization for anxiety disorders lasting for at least 4 days

^f^A participant discontinued the visits despite having an adequate treatment plan

^g^Fisher’s exact test was used in the analysis

^h^Psychotic, eating, sleeping, adjustment or impulse control disorder lifetime

^i^Participants with a single specific phobia were excluded

^j^A bivariate analysis

*The *p*-values indicate a significance of the difference between categories in a distribution of treatments and dropout tested by *χ*2- or Fisher’s exact test. *P*-values < 0.05 in boldface

**Table 6:** Sociodemographic factors, comorbid disorders, benzodiazepine use and misuse during the most recent treatment episode for anxiety disorders^c,^ ^d ^

			Benzodiazepine				Benzodiazepine misuse			
			Not prescribed		Prescribed		No		Yes	
Variable	Category		%	N	%	N	%	N	%	N
All			65.4	51	34.6	27	94.9	75	5.1	4
Gender	Male		50.0	11	50.0	11	95.5	21	4.6	1
	Female		71.4	40	28.6	16	94.7	54	5.3	3
		p*	0.073				1.000^a^			
Agegroup	<25 years		73.3	11	26.7	4	100.0	15	0.0	0
	25–29 years		69.7	23	30.3	10	97.1	33	2.9	1
	≥30 years		56.7	17	43.3	13	90.0	27	10.0	3
		p*	0.428				0.402^a^			
Basic	Less than high school		64.9	24	35.1	13	97.4	37	2.6	1
education	High school		71.1	27	29.0	11	94.7	36	5.3	2
		p*	0.566				1.000^a^			
Current	Employed		75.0	30	25.0	10	100.0	40	0.0	0
employment	Student		57.1	8	42.9	6	92.9	13	7.1	1
	Unemployed		55.6	5	44.4	4	100.0	9	0.0	0
	Other		66.7	8	33.3	4	84.6	11	15.4	2
		p*	0.485^a^				0.061^a^			
Married or	No		66.7	18	33.3	9	96.3	26	3.7	1
cohabiting	Yes		68.8	33	31.3	15	95.9	47	4.1	2
		p*	0.853				1.000^a^			
Comorbid	Yes		66.7	32	33.3	16	93.8	45	6.3	3
mood	No		63.3	19	36.7	11	96.8	30	3.2	1
disorder		p*	0.763				1.000^a^			
Comorbid	Yes		43.5	10	56.5	13	87.0	20	13.0	3
substance use	No		74.6	41	25.5	14	98.2	55	1.8	1
disorder		p*	**0.009**				0.072^a^			
Comorbid	Yes		31.6	6	68.4	13	80.0	16	20.0	4
personality	No		76.3	45	23.7	14	100.0	59	0.0	0
disorder		p*	**0.0004**				**0.003** ^a^			
Comorbid other	Yes		50.0	8	50.0	8	81.3	13	18.8	3
disorder^b^	No		69.4	43	30.7	19	98.4	62	1.6	1
		p*	0.147				**0.025** ^a^			
More than 1	Yes		73.3	11	26.7	4	87.5	14	12.5	2
anxiety	No		63.5	40	36.5	23	96.8	61	3.2	2
disorder		p*	0.472				0.181^a^			

^a^Fisher’s exact test was used in the analysis

^b^Psychotic, eating, sleeping, adjustment or impulse control disorder lifetime

^c^Participants with a single specific phobia were excluded

^d^A bivariate analysis

*The *p*-values indicate a significance of the difference between categories in a distribution of treatments and dropout tested by *χ*2- or Fisher’s exact test. *P*-values < 0.05 in boldface

Participants with comorbid personality disorders received and misused benzodiazepines more often than others according to the bivariate analysis (Table 6). An association was also found between personality disorders and visits with a physician at least four times within 12 months (Table 5).

No associations were found between comorbid disorders and treatment dropouts (Table 5).

### Factors associated with treatment in the multivariate analyses

In the multivariate analysis, comorbid substance use disorder (OR 4.48, CI 1.11–18.08, *P* = 0.035) was associated with antidepressant or buspirone medication lasting at least 2 months (Table 7). Comorbid personality disorder in turn was associated with increased odds for benzodiazepine medication (OR 4.77, CI 1.11–20.59, *P* = 0.036) (Table 8). Those who were currently married or cohabiting had lower odds of having at least four visits with a physician a year (OR 0.27, CI 0.08–0.85, *P* = 0.025) (Table 7). None of these factors explained the final outcome of minimally adequate treatment, but the *p*-value for the association between marital status and minimally adequate treatment was close to being significant (OR 0.36, CI 0.12–1.05, *P* = 0.062) and was significant when we studied the most intensive treatment episode (OR 0.33, CI 0.11–0.99, *P* = 0.048) (Table 7 and Additional file 5).

**Table 7:** Variables associated with treatments received during the most recent treatment period for anxiety disorders^g, h^

		Pharmacotherapy				Visits with a physician / a year				Guideline-concordant pharmacotherapy^c^		Sessions of psychotherapy / a year				Minimally adequate treatment^e^	
		Any^a^		≥2 months		Any^b^		≥4 times				Any^d^		≥8 times			
Variable	Category	OR	95 % CI	OR	95 % CI	OR	95 % CI	OR	95 % CI	OR	95 % CI	OR	95 % CI	OR	95 % CI	OR	95 % CI
Gender	Male (ref.)	1.00	-	1.00	-	1.00	-	1.00	-	1.00	-	1.00	-	1.00	-	1.00	-
	Female	1.19	0.34–4.14	1.06	0.29–3.90	1.95	0.50–7.64	1.16	0.30–4.56	0.72	0.18–2.87	1.18	0.31–4.47	1.50	0.38–5.92	1.18	0.33–4.18
Basic education	Less than high school (ref.)	1.00	-	1.00	-	1.00	-	1.00	-	1.00	-	1.00	-	1.00	-	1.00	-
	High school	1.33	0.42–4.18	3.08	0.86–11.04	1.42	0.41–4.97	0.92	0.25–3.35	1.57	0.40–6.09	1.04	0.31–3.45	1.12	0.32–3.94	0.71	0.22–2.30
Married or	No (ref.)	1.00	-	1.00	-	1.00	-	1.00	-	1.00	-	1.00	-	1.00	-	1.00	-
cohabiting	Yes	0.43	0.15–1.25	0.85	0.28–2.56	0.34	0.09–1.25	*0.27	**0.08–0.85**	0.66	0.20–2.11	0.35	0.11–1.14	0.39	0.12–1.21	0.36	0.12–1.05
Comorbid	No (ref.)	1.00	-	1.00	-	1.00	-	1.00	-	1.00	-	1.00	-	1.00	-	1.00	-
mood disorder	Yes	1.07	0.36–3.14	1.47	0.47–4.60	1.28	0.41–4.00	0.99	0.31–3.15	1.59	0.46–5.44	0.71	0.22–2.26	0.70	0.21–2.34	1.07	0.37–3.13
Comorbid substance	No (ref.)	1.00	-	1.00	-	1.00	-	1.00	-	1.00	-	1.00	-	1.00	-	1.00	-
use disorder	Yes	1.92	0.54–6.86	*4.48	**1.11–18.08**	2.46	0.59–10.30	1.46	0.39–5.48	1.78	0.46–6.95	3.21	0.81–12.75	1.27	0.33–4.82	0.98	0.28–3.42
Comorbid	No (ref.)	1.00	-	1.00	-	1.00	-	1.00	-	1.00	-	1.00	-	1.00	-	1.00	-
personality disorder	Yes	1.87	0.49–7.20	1.40	0.34–5.84	1.55	0.38–6.35	2.20	0.55–8.70	1.76	0.41–7.51	2.05	0.49–8.48	4.03	0.97–16.83	2.24	0.61–8.26
Comorbid	No (ref.)	1.00	-	1.00	-	1.00	-	1.00	-	1.00	-	1.00	-	1.00	-	1.00	-
other disorder^f^	Yes	0.89	0.23–3.41	0.51	0.12–2.12	0.26	0.06–1.10	2.36	0.59–9.45	1.48	0.35–6.22	0.43	0.11–1.77	1.02	0.25–4.11	1.24	0.32–4.72
More than 1 anxiety	No (ref.)	1.00	-	1.00	-	1.00	-	1.00	-	1.00	-	1.00	-	1.00	-	1.00	-
disorder	Yes	0.59	0.15–2.31	0.45	0.10–1.96	0.64	0.16–2.53	0.97	0.22–4.19	0.74	0.16–3.35	0.99	0.25–3.96	1.90	0.45–8.01	0.80	0.21–2.97

*OR* Adjusted odds ratio; *95 % CI* 95 % confidence interval

*p < 0.05; **p < 0.01; ***p < 0.001. These *p*-values indicate a significance of the difference of the odds ratios between categories tested by *χ*2-test. Significant differences (*p* < 0.05) in boldface

^a^Antidepressant or buspirone prescribed

^b^At least 1 visit with a physician a year

^c^Antidepressant or buspirone used for at least 2 months + 4 visits with a physician a year

^d^At least 1 session of psychotherapy a year

^e^Antidepressant or buspirone used for at least 2 months + at least 4 visits with a physician a year or at least 8 sessions of psychotherapy a year or a hospitalization for anxiety disorders lasting for at least 4 days

^f^Psychotic, eating, sleeping, adjustment or impulse control disorder, lifetime

^g^Participants with a single specific phobia were excluded

^h^All the variables were entered simultaneously into a logistic regression model, adjusting for the other factors shown in the table

**Table 8:** Variables associated with benzodiazepine use during the most recent treatment period for anxiety disorders^b, c^

		Benzodiazepine prescribed	
Variable	Category	OR	95 % CI
Gender	Male (ref.)	1.00	-
	Female	0.66	0.16–2.63
Basic education	Less than high school (ref.)	1.00	-
	High school	1.46	0.37–5.81
Married or	No (ref.)	1.00	-
cohabiting	Yes	0.94	0.29–3.08
Comorbid	No (ref.)	1.00	-
mood disorder	Yes	0.66	0.20–2.21
Comorbid substance	No (ref.)	1.00	-
use disorder	Yes	2.30	0.57–9.26
Comorbid	No (ref.)	1.00	-
personality disorder	Yes	*4.77	**1.11–20.59**
Comorbid	No (ref.)	1.00	-
other disorder^a^	Yes	1.72	0.39–7.47
More than 1 anxiety	No (ref.)	1.00	-
disorder	Yes	0.45	0.08–2.65

*OR* Adjusted odds ratio; *95 % CI* 95 % confidence interval

*p < 0.05; **p < 0.01; ***p < 0.001. These *p*-values indicate a significance of the difference of the odds ratios between categories tested by *χ*2-test. Significant differences (*p* < 0.05) in boldface

^a^Psychotic, eating, sleeping, adjustment or impulse control disorder lifetime

^b^Participants with a single specific phobia were excluded

^c^All the variables were entered simultaneously into a logistic regression model, adjusting for the other factors shown in the table

## Discussion

In our study of a nationally representative sample of young adults with anxiety disorder (excluding those with a single specific phobia), 70.9 % had some kind of contact with the health care system for their anxiety disorder, and 41.8 % had received minimally adequate treatment during the most recent treatment period. In the adult sample of the Health 2000 Survey, only 33.5 % of participants with anxiety disorders had used health care services for mental health problems during the preceding 12 months [8]. According to these figures young adults seek and receive more treatment than older adults in Finland. The same kind of trend was found in the treatment of depressive disorders in the Health 2000 Survey, where 40.9 % of young adults received minimally adequate treatment during the last depressive episode, whereas only 18 % of individuals with MDD aged over 30 had received minimally adequate treatment during the previous 12 months [27, 45].

A comparison with previous surveys concerning treatment adequacy is difficult because of the different settings and criteria for minimally adequate treatment and also a variation in diagnoses included in anxiety disorders. In the NCS-R-survey, where the criteria of minimally adequate treatment were very similar to ours, 33.8 % of treated patients (42.2 %) received minimally adequate treatment, corresponding to a treatment adequacy of about 14 % among all participants with anxiety disorders [44]. Fernandez et al. obtained quite similar results in Spain, where 30.7 % of treated patients (41.8 %) with any anxiety disorder received minimally adequate treatment, corresponding to about 13 % of all participants with anxiety disorders [16]. Compared to these figures, the situation is better for young adults in Finland. In a recent German study, the proportion of people with lifetime anxiety disorder with a self-reported mental health care contact was similar to ours, varying from 42.7 % for obsessive-compulsive disorder to 69.2 % in generalized anxiety disorder [13].

The higher proportion of adequate treatment in our study may partly be explained by our versatile information search, which also included medical records. The young age of our participants and the fact that we studied the most recent treatment episode also decreases memory bias in this study. We included telephone consultations by patient or another health care professional as visits with a physician, which may slightly increase the proportion of adequate treatment. Furthermore, we defined hospitalization lasting at least 4 days as adequate care. However, these cases were few (8 participants) and almost all (7 participants) had also received adequate treatment in outpatient care.

None of the factors chosen were related to the adequacy of treatment. However, currently married or cohabiting subjects had less often had physician visits in accordance with guidelines according to the multivariate analysis. In addition, those who were currently employed had less often had physician visits and psychotherapy in accordance with guidelines according to the bivariate analysis. These figures may suggest that among the participants with anxiety disorders there is a subgroup who has less severe illness, less disabilities, more social support and good abilities to help themselves. This is supported by the fact that anxiety disorders consist of several illnesses for which the clinical significance may vary. However, despite good social functioning, untreated illness may cause personal suffering and lead to disabilities.

Comorbid substance use disorder was related to both antidepressant or buspirone medication lasting at least 2 months and benzodiazepine use, and the first difference was statistically significant even after adjusting for other factors. Moreover, comorbid personality disorder was associated with benzodiazepine use in the multivariate analysis and additionally with benzodiazepine misuse and physician visits in accordance with guidelines in the bivariate analysis. So, there may be a trend to treat patients with complicated illness more intensively. Unfortunately this pursuit may sometimes lead to inappropriate use of benzodiazepines.

Benzodiazepines had been prescribed to 34.6 % of people with anxiety disorders, and benzodiazepine (BZD) use was strongly associated with having a comorbid personality or substance use disorder. BZD misuse was infrequent, but our figure was probably an underestimate since it was either based on self-report in the interview or diagnosed abuse/dependence by the treating physician. There is evidence of short-term effectiveness of BZDS in panic disorder, generalized anxiety disorder and to some extent in social phobia, but BZDs are not effective in obsessive-compulsive disorder or post-traumatic stress disorder, and there is no evidence of long-term effectiveness in any anxiety disorder [46]. BZDs are not recommended as a first-line treatment in any anxiety disorder because of the controversy over whether the short-term benefits outweigh the risk of dependence and side effects, which include daytime drowsiness, impairment of learning, psychomotor slowing and increased risk of accidents [46]. BZDs may also cause paradoxical reactions, including increased anxiety, agitation, hyperactivity and aggressiveness, and the risk of these is increased in people with personality and substance use disorders [46]. Therefore, it is alarming that BZD use was particularly common in people with personality and substance use disorders. The results suggest that more attention should be paid to prescription of BZDs to people with anxiety disorders in Finland.

In our study, 11.0 % of participants discontinued the visits, but none of the sociodemographic factors or comorbidities were related to dropout. This is in line with a review of Santana and Fontenelle, who found the prevalence of dropouts varied from 10.3 to 57.0 % in different studies. They also stated that no consistent conclusion can be reached regarding the effect of sociodemographic factors and clinical features on adherence of patients, though they emphasized the significance of cognitive variables, such as expectations and beliefs about the disease and its treatment, in managing patients with anxiety disorders [47].

The main strength of this study was a two-phase study design which enabled us to conduct SCID-I interviews, which were done by experienced mental health professionals. We also had access to case records from all mental health treatments, which compensated for any possible effect of recall bias in the final diagnostic assessment. Therefore we cannot directly compare our results with most previous surveys using only information collected by interviews. The percentage of minimally adequate treatment was slightly smaller (37.1 %) when we excluded those patients who had not been interviewed, for whom we only had medical records.

Regarding limitations, they have also been discussed in detail in our previous article which focused on the treatment of depressive disorders [27]. To summarize: attrition is the main limitation of a two-phase study design [48]. In this study, attrition, both in the questionnaire and the interview, was associated with age, gender, education and hospital treatment, but not with self-reported mental health disorders or symptoms at the baseline survey. A more detailed analysis of nonresponse has been reported previously [26].

Another notable limitation was a small size of the study sample, which led to low statistical power. This was also a reason why we could not analyze the treatments for each anxiety disorder separately. Moreover, correction for multiple testing was not done.

A minor limitation is that SCID-II was not used in the interview. The diagnostic assessment of personality disorders was based on other available systematically evaluated information from the interview and/or case records by experienced clinicians.

Psychotherapy in this study means psychosocial support broadly, because we did not have information on whether the mental health professional providing treatment was a licensed psychotherapist. Therefore, we cannot draw conclusions on the access to actual psychotherapy on the basis of this study.

The Mental Health in Early Adulthood in Finland (MEAF) study was carried out about 10 years ago. Based on official statistics, the use of antidepressants and the number of psychiatric outpatient visits has increased and also the availability of psychotherapy is better in Finland nowadays. Therefore, the figures of minimally adequate treatment may be better today.

## Conclusions

More efforts are needed to treat young adults with anxiety disorders, since less than a half of them received minimally adequate treatment, though more than 70 % seek treatment. Benzodiazepine use was more frequent among individuals with personality and substance use disorders, who may be especially vulnerable to their negative effects.

## Additional files

Additional file 1: Figure S1. Mental health in Early Adulthood in Finland (MEAF) study flow. (PDF 7 kb) Additional file 2: Table S1. Sociodemographic factors, treatments received and dropouts during the most intensive treatment episode for anxiety disorders. (DOC 95 kb) Additional file 3: Table S2. Comorbid disorders, treatments received and dropouts during the most intensive treatment episode for anxiety disorders. (DOC 82 kb) Additional file 4: Table S3. Sociodemographic factors, comorbid disorders, benzodiazepine use and misuse during the most intensive treatment episode for anxiety disorders. (DOC 96 kb) Additional file 5: Table S4. Variables associated with treatments received during the most intensive treatment period for anxiety disorders. (DOC 79 kb) Additional file 6: Table S5. Variables associated with benzodiazepine use during the most intensive treatment period for anxiety disorders. (DOC 48kb)
