# Supplementary material for: Treatment adequacy of anxiety disorders among young adults in Finland
Source: BMC Psychiatry. 2016 Mar 15;16:63. doi: 10.1186/s12888-016-0766-0 (PMC4799592; doi:10.1186/s12888-016-0766-0)

Additional file 1: Figure S1 Mental Health in Early Adulthood in Finland (MEAF) study flow

Adapted from Suvisaari et al. 2009, courtesy of Cambridge University Press.

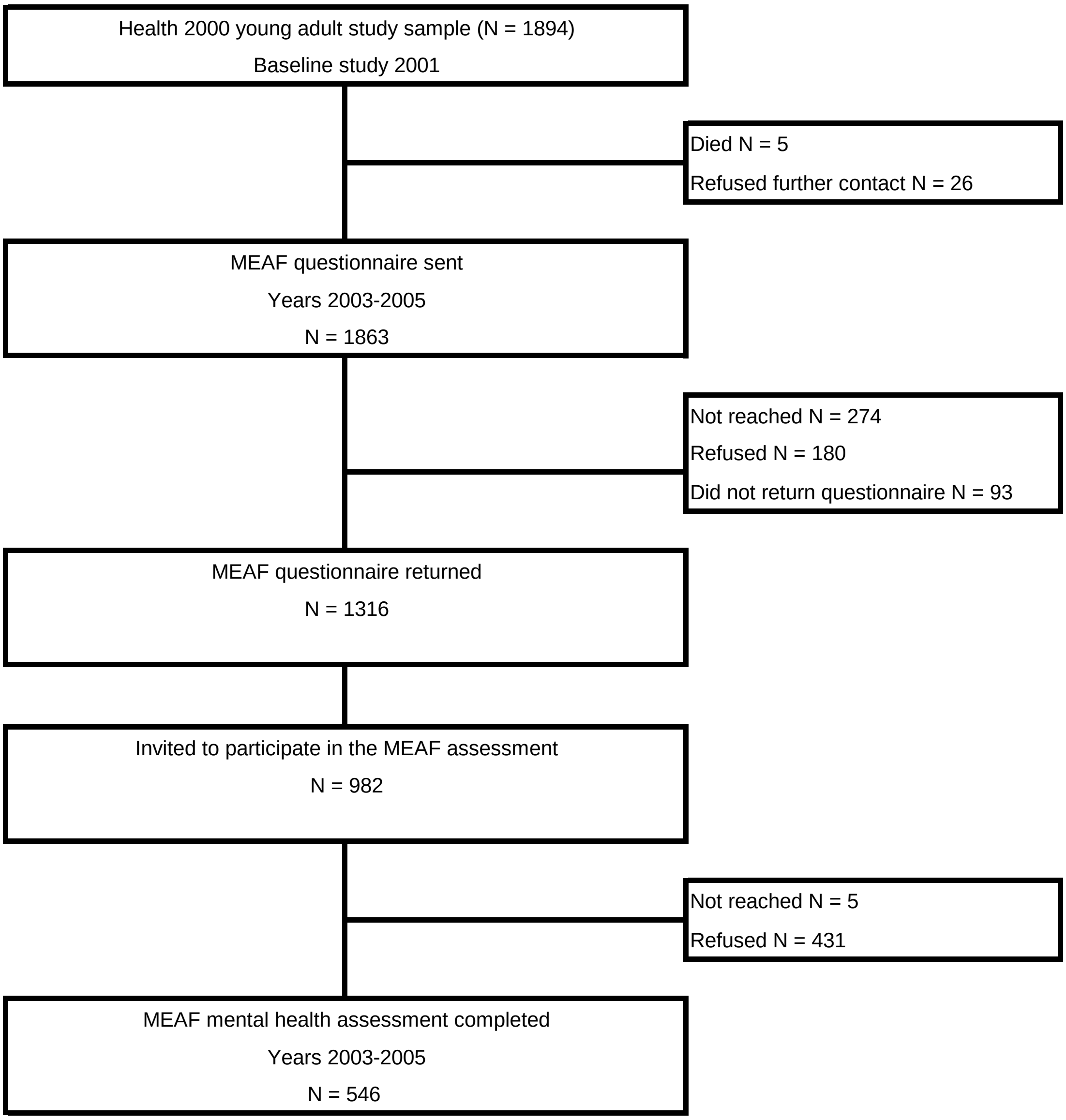

Supplement: Additional file 1: Figure S1. — Mental health in Early Adulthood in Finland (MEAF) study flow. (PDF 7 kb) [file 12888_2016_766_MOESM1_ESM.pdf]
