# Supplementary material for: Treatment adequacy of anxiety disorders among young adults in Finland
Source: BMC Psychiatry. 2016 Mar 15;16:63. doi: 10.1186/s12888-016-0766-0 (PMC4799592; doi:10.1186/s12888-016-0766-0)
Supplement: Additional file 2: Table S1. — Sociodemographic factors, treatments received and dropouts during the most intensive treatment episode for anxiety disorders. (DOC 95 kb) [file 12888_2016_766_MOESM2_ESM.doc]

**Additional file 2: Table S1 Sociodemographic factors, treatments received and dropouts during the most intensive treatment episode for anxiety disorders^i, j^**

|  |  |  |  | | | |  | | | | **Guideline concordant pharmacotherapy^c^** | | **Sessions of**  **psychotherapy / a year** | | | | **Minimally adequate treatment^e^** | | **Treatment dropout^f^** | |
| --- | --- | --- | --- | --- | --- | --- | --- | --- | --- | --- | --- | --- | --- | --- | --- | --- | --- | --- | --- | --- |
|  |  |  |  | | | | **Visits with** | | | |
|  |  |  | **Pharmacotherapy**  **Pharmacotherapy** | | | | **a physician** | | | |
|  |  |  | **Any^a^** | | **≥2 months** | | **Any^b^** | | **≥4 times** | |  | | **Any^d^** | | **≥8 times** | |  | |  | |
| **Variable** | **Category** |  | **%** | **N** | **%** | **N** | **%** | **N** | **%** | **N** | **%** | **N** | **%** | **N** | **%** | **N** | **%** | **N** | **%** | **N** |
| **All** |  |  | 48.1 | 38 | 39.7 | 31 | 67.5 | 52 | 40.3 | 31 | 27.9 | 22 | 60.3 | 47 | 38.5 | 30 | 48.1 | 38 | 16.0 | 12 |
| **Gender** | **Male** |  | 59.1 | 13 | 50.0 | 11 | 72.7 | 16 | 50.0 | 11 | 40.9 | 9 | 71.4 | 15 | 38.1 | 8 | 54.6 | 12 | 22.7 | 5 |
|  | **Female** |  | 43.9 | 25 | 35.7 | 20 | 65.5 | 36 | 36.4 | 20 | 22.8 | 13 | 56.1 | 32 | 38.6 | 22 | 45.6 | 26 | 13.2 | 7 |
|  |  | **p^g^** | 0.225 |  | 0.246 |  | 0.538 |  | 0.270 |  | 0.108 |  | 0.221 |  | 0.968 |  | 0.476 |  | 0.318^h^ |  |
| **Agegroup** | **<25 years** |  | 66.7 | 10 | 60.0 | 9 | 78.6 | 11 | 42.9 | 6 | 40.0 | 6 | 71.4 | 10 | 57.1 | 8 | 60.0 | 9 | 7.7 | 1 |
|  | **25-29 years** |  | 41.2 | 14 | 35.3 | 12 | 60.6 | 20 | 36.4 | 12 | 26.5 | 9 | 55.9 | 19 | 17.7 | 6 | 38.2 | 13 | 17.7 | 6 |
|  | **≥30 years** |  | 46.7 | 14 | 34.5 | 10 | 70.0 | 21 | 43.3 | 13 | 23.3 | 7 | 60.0 | 18 | 53.3 | 16 | 53.3 | 16 | 17.9 | 5 |
|  |  | **p^g^** | 0.253 |  | 0.203 |  | 0.453 |  | 0.833 |  | 0.487 |  | 0.606 |  | **0.004** |  | 0.286 |  | 0.836^h^ |  |
| **Basic** | **Less than high school** |  | 42.1 | 16 | 34.2 | 13 | 63.9 | 23 | 38.9 | 14 | 26.3 | 10 | 63.2 | 24 | 42.1 | 16 | 55.3 | 21 | 20.0 | 7 |
| **education** | **High school** |  | 50.0 | 19 | 43.2 | 16 | 68.4 | 26 | 36.8 | 14 | 26.3 | 10 | 54.1 | 20 | 32.4 | 12 | 36.8 | 14 | 10.8 | 4 |
|  |  | **p^g^** | 0.490 |  | 0.422 |  | 0.680 |  | 0.856 |  | 1.000 |  | 0.423 |  | 0.387 |  | 0.107 |  | 0.279 |  |
| **Current** | **Employed** |  | 40.0 | 16 | 35.9 | 14 | 62.5 | 25 | 27.5 | 11 | 17.5 | 7 | 50.0 | 20 | 17.5 | 7 | 32.5 | 13 | 18.4 | 7 |
| **employment** | **Student** |  | 71.4 | 10 | 57.1 | 8 | 71.4 | 10 | 57.1 | 8 | 42.9 | 6 | 69.2 | 9 | 53.9 | 7 | 50.0 | 7 | 21.4 | 3 |
|  | **Unemployed** |  | 44.4 | 4 | 44.4 | 4 | 62.5 | 5 | 62.5 | 5 | 44.4 | 4 | 66.7 | 6 | 55.6 | 5 | 66.7 | 6 | 12.5 | 1 |
|  | **Other** |  | 38.5 | 5 | 23.1 | 3 | 75.0 | 9 | 33.3 | 4 | 23.1 | 3 | 69.2 | 9 | 69.2 | 9 | 69.2 | 9 | 0.0 | 0 |
|  |  | **p^g^** | 0.222^h^ |  | 0.308 |  | 0.853^h^ |  | 0.106^h^ |  | 0.147^h^ |  | 0.445 |  | **0.001^h^** |  | 0.059^h^ |  | 0.403^h^ |  |
| **Married or** | **No** |  | 63.0 | 17 | 46.2 | 12 | 80.8 | 21 | 57.7 | 15 | 37.0 | 10 | 69.2 | 18 | 46.2 | 12 | 59.3 | 16 | 19.2 | 5 |
| **cohabiting** | **Yes** |  | 36.7 | 18 | 34.7 | 17 | 58.3 | 28 | 27.1 | 13 | 20.4 | 10 | 53.1 | 26 | 32.7 | 16 | 38.8 | 19 | 13.0 | 6 |
|  |  | **p^g^** | **0.028** |  | 0.332 |  | 0.051 |  | **0.010** |  | 0.115 |  | 0.176 |  | 0.250 |  | 0.086 |  | 0.511^h^ |  |

^a^ Antidepressant or buspirone prescribed

^b^ At least 1 visit with a physician a year

^c^ Antidepressant or buspirone used for at least 2 months + 4 visits with a physician a year

^d^ At least 1 session of psychotherapy a year

^e^ Antidepressant or buspirone used for at least 2 months + at least 4 visits with a physician a year or at least 8 sessions of psychotherapy a year or a hospitalization for anxiety disorders lasting for at least 4 days

^f^ A participant discontinued the visits despite having an adequate treatment plan

^g^ The p-values indicate a significance of the difference between categories in a distribution of treatments and dropout tested by χ2- or Fisher's exact test. P-values < 0.05 in boldface

^h^ Fisher's exact test was used in the analysis

^i^ Participants with a single specific phobia were excluded

^j^ A bivariate analysis
