# Supplementary material for: Treatment adequacy of anxiety disorders among young adults in Finland
Source: BMC Psychiatry. 2016 Mar 15;16:63. doi: 10.1186/s12888-016-0766-0 (PMC4799592; doi:10.1186/s12888-016-0766-0)
Supplement: Additional file 3: Table S2. — Comorbid disorders, treatments received and dropouts during the most intensive treatment episode for anxiety disorders. (DOC 82 kb) [file 12888_2016_766_MOESM3_ESM.doc]

**Additional file 3: Table S2 Comorbid disorders, treatments received and dropouts during the most intensive treatment episode for anxiety disorders^j, k^**

|  |  |  |  | | | |  | | | | **Guideline concordant pharmacotherapy^c^** | | **Sessions of**  **psychotherapy / a year** | | | | **Minimally adequate treatment^e^** | | **Treatment dropout^f^** | |
| --- | --- | --- | --- | --- | --- | --- | --- | --- | --- | --- | --- | --- | --- | --- | --- | --- | --- | --- | --- | --- |
|  |  |  |  | | | | **Visits with** | | | |
|  |  |  | **Pharmacotherapy**  **Pharmacotherapy** | | | | **a physician** | | | |
|  |  |  | **Any^a^** | | **≥2 months** | | **Any^b^** | | **≥4 times** | |  | | **Any^d^** | | **≥8 times** | |  | |  | |
| **Variable** | **Category** |  | **%** | **N** | **%** | **N** | **%** | **N** | **%** | **N** | **%** | **N** | **%** | **N** | **%** | **N** | **%** | **N** | **%** | **N** |
| **Comorbid** | **Yes** |  | 52.1 | 25 | 41.7 | 20 | 71.7 | 33 | 47.8 | 22 | 33.3 | 16 | 60.4 | 29 | 39.6 | 19 | 52.1 | 25 | 22.2 | 10 |
| **mood** | **No** |  | 41.9 | 13 | 36.7 | 11 | 61.3 | 19 | 29.0 | 9 | 19.4 | 6 | 60.0 | 18 | 36.7 | 11 | 41.9 | 13 | 6.7 | 2 |
| **disorders** |  | **p^g^** | 0.378 |  | 0.661 |  | 0.337 |  | 0.099 |  | 0.176 |  | 0.971 |  | 0.797 |  | 0.378 |  | 0.108^h^ |  |
| **Comorbid** | **Yes** |  | 65.2 | 15 | 59.1 | 13 | 78.3 | 18 | 56.5 | 13 | 43.5 | 10 | 78.3 | 18 | 43.5 | 10 | 56.5 | 13 | 13.6 | 3 |
| **substance use** | **No** |  | 41.1 | 23 | 32.1 | 18 | 63.0 | 34 | 33.3 | 18 | 21.4 | 12 | 52.7 | 29 | 36.4 | 20 | 44.6 | 25 | 17.0 | 9 |
| **disorder** |  | **p^g^** | 0.051 |  | **0.029** |  | 0.190 |  | 0.058 |  | **0.047** |  | **0.036** |  | 0.556 |  | 0.337 |  | 1.000^h^ |  |
| **Comorbid** | **Yes** |  | 65.0 | 13 | 45.0 | 9 | 75.0 | 15 | 60.0 | 12 | 40.0 | 8 | 75.0 | 15 | 60.0 | 12 | 70.0 | 14 | 25.0 | 5 |
| **personality** | **No** |  | 42.4 | 25 | 37.9 | 22 | 64.9 | 37 | 33.3 | 19 | 23.7 | 14 | 55.2 | 32 | 31.0 | 18 | 40.7 | 24 | 12.7 | 7 |
| **disorder** |  | **p^g^** | 0.080 |  | 0.578 |  | 0.407 |  | **0.036** |  | 0.161 |  | 0.118 |  | **0.022** |  | **0.023** |  | 0.284^h^ |  |
| **Comorbid** | **Yes** |  | 50.0 | 8 | 40.0 | 6 | 56.3 | 9 | 56.3 | 9 | 37.5 | 6 | 50.0 | 8 | 43.8 | 7 | 50.0 | 8 | 12.5 | 2 |
| **other disorder^i^**  **disorder**  **disorder^i^** | **No** |  | 47.6 | 30 | 39.7 | 25 | 70.5 | 43 | 36.1 | 22 | 25.4 | 16 | 62.9 | 39 | 37.1 | 23 | 47.6 | 30 | 17.0 | 10 |
|  |  | **p^g^** | 0.865 |  | 0.982 |  | 0.279 |  | 0.143 |  | 0.360^h^ |  | 0.347 |  | 0.626 |  | 0.865 |  | 1.000^h^ |  |
| **More than 1** | **Yes** |  | 37.5 | 6 | 31.3 | 5 | 64.3 | 9 | 42.9 | 6 | 25.0 | 4 | 62.5 | 10 | 56.3 | 9 | 56.3 | 9 | 6.7 | 1 |
| **anxiety** | **No** |  | 50.8 | 32 | 41.9 | 26 | 68.3 | 43 | 39.7 | 25 | 28.6 | 18 | 59.7 | 37 | 33.9 | 21 | 46.0 | 29 | 18.3 | 11 |
| **disorder** |  | **p^g^** | 0.342 |  | 0.436 |  | 0.762^h^ |  | 0.827 |  | 1.000^h^ |  | 0.837 |  | 0.101 |  | 0.465 |  | 0.440^h^ |  |

^a^ Antidepressant or buspirone prescribed

^b^ At least 1 visit with a physician a year

^c^ Antidepressant or buspirone used for at least 2 months + 4 visits with a physician a year

^d^ At least 1 session of psychotherapy a year

^e^ Antidepressant or buspirone used for at least 2 months + at least 4 visits with a physician a year or at least 8 sessions of psychotherapy a year or a hospitalization for anxiety disorders lasting for at least 4 days

^f^ A participant discontinued the visits despite having an adequate treatment plan

^g^ The p-values indicate a significance of the difference between categories in a distribution of treatments and dropout tested by χ2- or Fisher's exact test. P-values < 0.05 in boldface

^h^ Fisher's exact test was used in the analysis

^i^ Psychotic, eating, sleeping, adjustment or impulse control disorders lifetime

^j^ Participants with a single specific phobia were excluded

^k^ A bivariate analysis
