# Supplementary material for: Treatment adequacy of anxiety disorders among young adults in Finland
Source: BMC Psychiatry. 2016 Mar 15;16:63. doi: 10.1186/s12888-016-0766-0 (PMC4799592; doi:10.1186/s12888-016-0766-0)
Supplement: Additional file 4: Table S3. — Sociodemographic factors, comorbid disorders, benzodiazepine use and misuse during the most intensive treatment episode for anxiety disorders. (DOC 96 kb) [file 12888_2016_766_MOESM4_ESM.doc]

**Additional file 4: Table S3 Sociodemographic factors, comorbid disorders, benzodiazepine use and misuse during the most intensive treatment episode for anxiety disorders^d,e^**

|  |  |  |  | | | |  | | | |
| --- | --- | --- | --- | --- | --- | --- | --- | --- | --- | --- |
|  |  |  | **Benzodiazepine** | | | | **Benzodiazepine misuse** | | | |
|  |  |  |  | | | |  | | | |
|  |  |  | **Not prescribed** | | **Prescribed** | | **No** | | **Yes** | |
| **Variable** | **Category** |  | **%** | **N** | **%** | **N** | **%** | **N** | **%** | **N** |
| **All** |  |  | 65.8 | 52 | 34.2 | 27 | 96.2 | 76 | 3.8 | 3 |
| **Gender** | **Male** |  | 50.0 | 11 | 50.0 | 11 | 100.0 | 22 | 0.0 | 0 |
|  | **Female** |  | 71.9 | 41 | 28.1 | 16 | 94.7 | 54 | 5.3 | 3 |
|  |  | **p^a^** | 0.066 |  |  |  | 0.556^b^ |  |  |  |
| **Agegroup** | **<25 years** |  | 73.3 | 11 | 26.7 | 4 | 100.0 | 15 | 0.0 | 0 |
|  | **25-29 years** |  | 70.6 | 24 | 29.4 | 10 | 97.1 | 33 | 2.9 | 1 |
|  | **≥30 years** |  | 56.7 | 17 | 43.3 | 13 | 93.3 | 28 | 6.7 | 2 |
|  |  | **p^a^** | 0.399 |  |  |  | 0.594^b^ |  |  |  |
| **Basic** | **Less than high school** |  | 65.8 | 25 | 34.2 | 13 | 97.4 | 37 | 2.6 | 1 |
| **education** | **High school** |  | 71.1 | 27 | 29.0 | 11 | 94.7 | 36 | 5.3 | 2 |
|  |  | **p^a^** | 0.622 |  |  |  | 1.000^b^ |  |  |  |
| **Current** | **Employed** |  | 75.0 | 30 | 25.0 | 10 | 100.0 | 40 | 0.0 | 0 |
| **employment** | **Student** |  | 57.1 | 8 | 42.9 | 6 | 92.9 | 13 | 7.1 | 1 |
|  | **Unemployed** |  | 55.6 | 5 | 44.4 | 4 | 100.0 | 9 | 0.0 | 0 |
|  | **Other** |  | 69.2 | 9 | 30.8 | 4 | 84.6 | 11 | 15.4 | 2 |
|  |  | **p^a^** | 0.490 |  |  |  | 0.061^b^ |  |  |  |
| **Married or** | **No** |  | 66.7 | 18 | 33.3 | 9 | 96.3 | 26 | 3.7 | 1 |
| **cohabiting** | **Yes** |  | 69.4 | 34 | 30.6 | 15 | 95.9 | 47 | 4.1 | 2 |
|  |  | **p^a^** | 0.807 |  |  |  | 1.000^b^ |  |  |  |
| **Comorbid** | **Yes** |  | 66.7 | 32 | 33.3 | 16 | 95.8 | 46 | 4.2 | 2 |
| **mood** | **No** |  | 64.5 | 20 | 35.5 | 11 | 96.8 | 30 | 3.2 | 1 |
| **disorder** |  | **p^a^** | 0.844 |  |  |  | 1.000^b^ |  |  |  |
| **Comorbid** | **Yes** |  | 43.5 | 10 | 56.5 | 13 | 91.3 | 21 | 8.7 | 2 |
| **substance use** | **No** |  | 75.0 | 42 | 25.0 | 14 | 98.2 | 55 | 1.8 | 1 |
| **disorder** |  | **p^a^** | **0.007** |  |  |  | 0.202^b^ |  |  |  |
| **Comorbid** | **Yes** |  | 35.0 | 7 | 65.0 | 13 | 85.0 | 17 | 15.0 | 3 |
| **personality** | **No** |  | 76.3 | 45 | 23.7 | 14 | 100.0 | 59 | 0.0 | 0 |
| **disorder** |  | **p^a^** | **0.001** |  |  |  | **0.014^b^** |  |  |  |
| **Comorbid other** | **Yes** |  | 50.0 | 8 | 50.0 | 8 | 81.3 | 13 | 18.8 | 3 |
| **disorder^c^** | **No** |  | 69.8 | 44 | 30.2 | 19 | 100.0 | 63 | 0.0 | 0 |
|  |  | **p^a^** | 0.135 |  |  |  | **0.007^b^** |  |  |  |
| **More than 1** | **Yes** |  | 75.0 | 12 | 25.0 | 4 | 93.8 | 15 | 6.3 | 1 |
| **anxiety** | **No** |  | 63.5 | 40 | 36.5 | 23 | 96.8 | 61 | 3.2 | 2 |
| **disorder** |  | **p^a^** | 0.386 |  |  |  | 0.498^b^ |  |  |  |

^a^ The p-values indicate a significance of the difference between categories in a distribution of treatments and

dropout tested by χ2- or Fisher's exact test. P-values < 0.05 in boldface

^b^ Fisher's exact test was used in the analysis

^c^ Psychotic, eating, sleeping, adjustment or impulse control disorder, lifetime

^d^ Participants with a single specific phobia were excluded

^e^ A bivariate analysis
