# Supplementary material for: Treatment adequacy of anxiety disorders among young adults in Finland
Source: BMC Psychiatry. 2016 Mar 15;16:63. doi: 10.1186/s12888-016-0766-0 (PMC4799592; doi:10.1186/s12888-016-0766-0)
Supplement: Additional file 5: Table S4. — Variables associated with treatments received during the most intensive treatment period for anxiety disorders. (DOC 79 kb) [file 12888_2016_766_MOESM5_ESM.doc]

**Additional file 5: Table S4 Variables associated with treatments received during the most intensive treatment period for anxiety disorders^g, h^**

|  |  |  | |  | | |  | | |  | |  | |  | |  | |  | **Guideline-** | |  | |  | |  | |  | **Minimally** | |
| --- | --- | --- | --- | --- | --- | --- | --- | --- | --- | --- | --- | --- | --- | --- | --- | --- | --- | --- | --- | --- | --- | --- | --- | --- | --- | --- | --- | --- | --- |
|  |  |  |  | | |  | | |  | | | **Visits with** | | | | | | | **concordant** | | **Sessions of** | | | | | | | **adequate** | |
|  |  | **Pharmacotherapy** | | | | | | | | | | **a physician / a year** | | | | | | | **pharmacotherapy^c^** | | **psychotherapy / a year** | | | | | | | **treatment^e^** | |
|  |  | **Any^a^** | | | | | | **≥2 months** | | | | **Any^b^** | | | **≥4 times** | | | |  | | **Any^d^** | | | **≥8 times** | | | |  | |
| **Variable** | **Category** | **OR** | | | **95% CI** | | | **OR** | | | **95% CI** | **OR** | **95% CI** | | **OR** | | **95% CI** | | **OR** | **95% CI** | **OR** | **95% CI** | | **OR** | | **95% CI** | | **OR** | **95% CI** |
| **Gender** | **Male (ref.)** | 1.00 | | | - | | | 1.00 | | | - | 1.00 | - | | 1.00 | | - | | 1.00 | - | 1.00 | - | | 1.00 | | - | | 1.00 | - |
|  | **Female** | 1.09 | | | 0.30-3.99 | | | 1.01 | | | 0.27-3.71 | 1.68 | 0.43-6.63 | | 1.05 | | 0.28-3.99 | | 0.62 | 0.16-2.40 | 1.32 | 0.35-4.97 | | 1.77 | | 0.46-6.84 | | 1.34 | 0.38-4.73 |
| **Basic education** | **Less than high school (ref.)** | 1.00 | | | - | | | 1.00 | | | - | 1.00 | - | | 1.00 | | - | | 1.00 | - | 1.00 | - | | 1.00 | | - | | 1.00 | - |
|  | **High school** | 2.48 | | | 0.71-8.63 | | | 2.79 | | | 0.79-9.90 | 2.07 | 0.57-7.47 | | 0.94 | | 0.26-3.35 | | 1.34 | 0.35-5.14 | 1.08 | 0.34-3.46 | | 0.77 | | 0.23-2.54 | | 0.43 | 0.13-1.36 |
| **Married or** | **No (ref.)** | 1.00 | | | - | | | 1.00 | | | - | 1.00 | - | | 1.00 | | - | | 1.00 | - | 1.00 | - | | 1.00 | | - | | 1.00 | - |
| **cohabiting** | **Yes** | 0.38 | | | 0.13-1.13 | | | 0.72 | | | 0.24-2.18 | 0.34 | 0.09-1.23 | | ***0.30** | | **0.10-0.90** | | 0.57 | 0.18-1.79 | 0.41 | 0.13-1.33 | | 0.42 | | 0.13-1.32 | | ***0.33** | **0.11-0.99** |
| **Comorbid** | **No (ref.)** | 1.00 | | | - | | | 1.00 | | | - | 1.00 | - | | 1.00 | | - | | 1.00 | - | 1.00 | - | | 1.00 | | - | | 1.00 | - |
| **mood disorder** | **Yes** | 1.36 | | | 0.45-4.06 | | | 1.28 | | | 0.42-3.94 | 1.47 | 0.47-4.58 | | 1.38 | | 0.45-4.27 | | 1.82 | 0.53-6.27 | 0.73 | 0.24-2.23 | | 0.61 | | 0.19-1.92 | | 0.83 | 0.28-2.44 |
| **Comorbid substance**  **s** | **No (ref.)** | 1.00 | | | - | | | 1.00 | | | - | 1.00 | - | | 1.00 | | - | | 1.00 | - | 1.00 | - | | 1.00 | | - | | 1.00 | - |
| **use disorder** | **Yes** | 2.97 | | | 0.78-11.36 | | | ***4.63** | | | **1.16-18.49** | 2.96 | 0.70-12.58 | | 1.70 | | 0.46-6.25 | | 2.11 | 0.55-8.05 | 2.95 | 0.75-11.62 | | 1.02 | | 0.27-3.77 | | 0.85 | 0.24-2.95 |
| **Comorbid** | **No (ref.)** | 1.00 | | | - | | | 1.00 | | | - | 1.00 | - | | 1.00 | | - | | 1.00 | - | 1.00 | - | | 1.00 | | - | | 1.00 | - |
| **personality disorder** | **Yes** | 2.67 | | | 0.68-10.54 | | | 1.37 | | | 0.34-5.60 | 1.95 | 0.47-8.09 | | 1.77 | | 0.45-6.89 | | 1.42 | 0.33-6.06 | 2.19 | 0.55-8.66 | | 3.57 | | 0.91-13.99 | | 2.15 | 0.57-8.16 |
| **Comorbid** | **No (ref.)** | 1.00 | | | - | | | 1.00 | | | - | 1.00 | - | | 1.00 | | - | | 1.00 | - | 1.00 | - | | 1.00 | | - | | 1.00 | - |
| **other disorder^f^** | **Yes** | 0.50 | | | 0.12-2.11 | | | 0.59 | | | 0.14-2.48 | 0.24 | 0.05-1.07 | | 1.96 | | 0.50-7.77 | | 1.45 | 0.34-6.18 | 0.37 | 0.09-1.51 | | 1.04 | | 0.26-4.11 | | 1.11 | 0.29-4.26 |
| **More than 1 anxiety** | **No (ref.)** | 1.00 | | | - | | | 1.00 | | | - | 1.00 | - | | 1.00 | | - | | 1.00 | - | 1.00 | - | | 1.00 | | - | | 1.00 | - |
| **disorder** | **Yes** | 0.34 | | | 0.08-1.42 | | | 0.49 | | | 0.12-2.04 | 0.54 | 0.13-2.28 | | 0.76 | | 0.17-3.31 | | 0.60 | 0.13-2.79 | 0.99 | 0.27-3.64 | | 2.77 | | 0.72-10.64 | | 1.35 | 0.37-4.94 |

*p<0.05; **p<0.01; ***p<0.001. These p-values indicate a significance of the difference of the odds ratios between categories tested by χ2-test. Significant differences (p<0.05) in boldface

^a^ Antidepressant or buspirone prescribed

^b^ At least 1 visit with a physician a year

^c^ Antidepressant or buspirone used for at least 2 months + 4 visits with a physician a year

^d^ At least 1 session of psychotherapy a year

^e^ Antidepressant or buspirone used for at least 2 months + at least 4 visits with a physician a year or at least 8 sessions of psychotherapy a year or a hospitalization for anxiety disorders lasting for at least 4 days

^f^ Psychotic, eating, sleeping, adjustment or impulse control disorder, lifetime

^g^ Participants with a single specific phobia were excluded

^h^ All the variables were entered simultaneously into a logistic regression model, adjusting for the other factors shown in the table

OR = Adjusted odds ratio; 95% CI = 95% confidence interval
