# Supplementary material for: Treatment adequacy of anxiety disorders among young adults in Finland
Source: BMC Psychiatry. 2016 Mar 15;16:63. doi: 10.1186/s12888-016-0766-0 (PMC4799592; doi:10.1186/s12888-016-0766-0)
Supplement: Additional file 6: Table S5. — Variables associated with benzodiazepine use during the most intensive treatment period for anxiety disorders. (DOC 48kb) [file 12888_2016_766_MOESM6_ESM.doc]

**Additional file 6: Table S5 Variables associated with benzodiazepine**

**use during the most intensive treatment period for anxiety disorders^b,c^**

|  |  | **Benzodiazepine**  **prescribed** | |
| --- | --- | --- | --- |
|  |  |  |  |
|  |  |  |  |
|  |  |  |  |
| **Variable** | **Category** | **OR** | **95% CI** |
| **Gender** | **Male (ref.)** | 1.00 | - |
|  | **Female** | 0.63 | 0.16-2.50 |
| **Basic education** | **Less than high school (ref.)** | 1.00 | - |
|  | **High school** | 1.50 | 0.37-6.04 |
| **Married or** | **No (ref.)** | 1.00 | - |
| **cohabiting** | **Yes** | 0.94 | 0.29-3.08 |
| **Comorbid** | **No (ref.)** | 1.00 | - |
| **mood disorder** | **Yes** | 0.73 | 0.22-2.35 |
| **Comorbid substance**  **s** | **No (ref.)** | 1.00 | - |
| **use disorder** | **Yes** | 2.39 | 0.60-9.56 |
| **Comorbid** | **No (ref.)** | 1.00 | - |
| **personality disorder** | **Yes** | ***4.32** | **1.03-18.14** |
| **Comorbid** | **No (ref.)** | 1.00 | - |
| **other disorder^a^** | **Yes** | 1.76 | 0.41-7.65 |
| **More than 1 anxiety** | **No (ref.)** | 1.00 | - |
| **disorder** | **Yes** | 0.36 | 0.07-1.96 |

*p<0.05; **p<0.01; ***p<0.001. These p-values indicate a significance of the difference.

of the odds ratios between categories tested by χ2-test. Significant differences (p<0.05) in boldface

^a^ Psychotic, eating, sleeping, adjustment or impulse control disorder, lifetime

^b^ Participants with a single specific phobia were excluded

^c^ All the variables were entered simultaneously into a logistic regression model,

adjusting for the other factors shown in the table

OR = Adjusted odds ratio; 95% CI = 95% confidence interval
